# Caregiving Intensity and Psychosocial Impact of COVID-19 in Dementia and Non-Dementia Caregivers

**DOI:** 10.1093/geroni/igab046.2946

**Published:** 2021-12-17

**Authors:** Marika Humber, Angeline Truong, Madhuvanthi Suresh, Josephine Jacobs, Sam Thomas, Trevor Lee, Rashmi Risbud, Ranak Trivedi

**Affiliations:** 1 VA Palo Alto Healthcare System, Menlo Park, California, United States; 2 Stanford University School of Medicine, Stanford, California, United States; 3 Palo Alto University, Atlanta, Georgia, United States; 4 VA Palo Alto Health Care System, Menlo Park, California, United States; 5 Stanford School of Medicine, Stanford, California, United States; 6 VA Palo Alto Health Care System, VA Palo Alto Health Care System, California, United States; 7 VA Palo Alto Health Care System, Palo Alto, California, United States; 8 Stanford University, Menlo Park, California, United States

## Abstract

COVID-19 has adversely impacted the well-being of informal caregivers (CG) due to infection risk, changes to the home environment, and changes to resource availability. CG of persons living with dementia (PLWD) may be especially vulnerable due to the intensity of care provided. We compared CG activities and well-being among CG who did and did not care for PLWD during COVID-19. We conducted an anonymous online survey from April 2020-present. Respondents self-identified as 18+ years and CG to a child or adult with mental health or medical conditions. CG answered questions regarding hours of care provision and caregiving activities, and completed measures of CG burden (Zarit Burden Inventory-4), loneliness (UCLA Loneliness Scale), depressive symptoms (Patient Health Questionnaire), and anxiety (Generalized Anxiety Disorder-2). Of the 258 respondents within the United States, 86 cared for PLWD (33%; 88% female; 56±12 years) while 172 did not (66%; 87% female; 49±14 years). Compared to non-dementia CGs, more CGs of PLWD provided 40+ hours of caregiving/week (36% vs. 49%, p<.05), performed more caregiving activities (8.5 vs. 10.5, p<.01), and assisted with more activities of daily living (55% vs. 73%, p<.01). Compared to non-dementia CG, more dementia CG reported CG burden (53% vs. 67%; p<.05) and loneliness (7.3 vs. 9.1, p<.05). No differences in depressive symptoms or anxiety were found. Results suggest that existing needs of CG of PLWD may be exacerbated by the stressors and concerns of the pandemic, necessitating higher levels of support.

